# Combination therapy with toripalimab and lenvatinib in metastatic type 2 papillary renal cell carcinoma: a Case Report

**DOI:** 10.3389/fimmu.2025.1591489

**Published:** 2025-11-06

**Authors:** Wende Wang, Minna Chen, Wenwu Xue, De Zeng

**Affiliations:** The Department of Medical Oncology, Cancer Hospital of Shantou University Medical College, Shantou, China

**Keywords:** type 2 papillary renal cell carcinoma, immunotherapy, toripalimab, lenvatinib, p504s

## Abstract

**Background:**

Type 2 papillary renal cell carcinoma (PRCC) is an aggressive subtype of renal cell carcinoma with a poor prognosis. Diagnosis is challenging, particularly when presenting as a metastatic lung mass. This report describes a case of metastatic type 2 PRCC treated with first-line toripalimab (anti-PD-1) and lenvatinib (multikinase inhibitor), highlighting sustained clinical benefit.

**Case presentation:**

A 73-year-old man presented with cough, hemoptysis, and a large left lung mass initially misdiagnosed as primary lung cancer. Multidisciplinary re-evaluation, including immunohistochemistry (PAX8+, P504s+, also known as AMACR), confirmed metastatic type 2 PRCC. After 11 months of toripalimab (flat dose of 240 mg every 3 weeks) and lenvatinib (flat dose of 8 mg daily), serial imaging demonstrated partial response (PR) with regression of pulmonary metastases, lymphadenopathy, and obstructive pneumonia. No serious adverse events occurred.

**Conclusions:**

This case underscores the potential efficacy and tolerability of toripalimab-lenvatinib combination therapy in metastatic type 2 PRCC. Further clinical trials are warranted to validate this approach.

## 1 Introduction

Type 2 papillary renal cell carcinoma (PRCC) accounts for 10–15% of renal cell carcinomas and is distinguished by aggressive behavior, frequent metastasis, and poor prognosis (1). Unlike type 1 PRCC, type 2 tumors often exhibit rapid progression and resistance to conventional therapies (2). Metastatic type 2 PRCC poses diagnostic challenges, particularly when presenting as a lung mass mimicking primary pulmonary malignancy (3).

Therapeutic advances in renal cell carcinoma include tyrosine kinase inhibitor (TKI) such as lenvatinib, which targets VEGF receptors, and immune checkpoint inhibitor (ICI) like toripalimab (4, 5). Preclinical and clinical studies suggest synergy between TKI and immunotherapy, but data specific to type 2 PRCC remain limited (6). This report details a case of metastatic type 2 PRCC achieving sustained response to toripalimab-lenvatinib therapy, emphasizing the need for further investigation.

## 2 Case presentation

A 73-year-old man presented in May 2023 with cough and hemoptysis. Chest CT revealed a left lung mass. Initial biopsies suggested sarcomatoid carcinoma, but the patient declined chemotherapy. By December 2023, he developed a productive cough, worsening dyspnea, lower limb edema, and pleural effusions. Re-evaluation at Shantou Shengbao Cerebrovascular Disease Hospital identified mediastinal lymphadenopathy and bilateral lung nodules. The patient presented no significant medical history and no family history of relevant disorders or malignancies.

**Figure 1:**
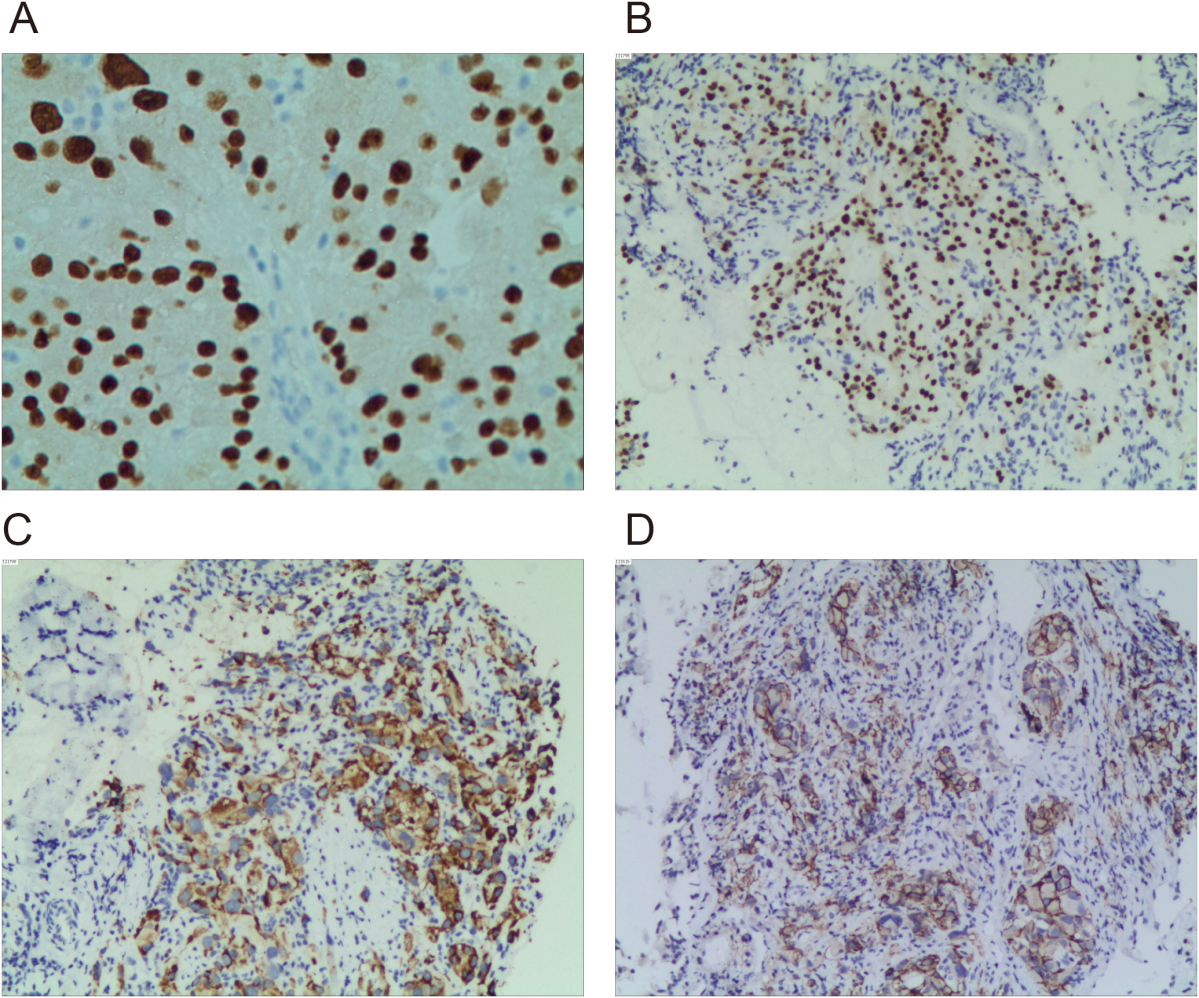
Immunohistochemical staining images of PAX8, P504s, and PD-L1. **(A)** Immunohistochemical detection of PAX8 in left supraclavicular lymph node specimens (×400). **(B)** Immunohistochemical detection of PAX8 in left upper lobe specimen (×200). **(C)** Immunohistochemical detection of P504s in left upper lobe specimen (×200). **(D)** Immunohistochemical detection of PD-L1 in left upper lobe specimen (×200).

In early January 2024, the patient presented to our hospital for medical evaluation with a Karnofsky Performance Status (KPS) score of 50. Immunohistochemistry (IHC) of a supraclavicular lymph node biopsy revealed PAX8++ and P504s++, consistent with metastatic renal cell carcinoma (**Figure 1A**). Further IHC of the lung mass confirmed type 2 PRCC (PAX8++, P504s+++) (**Figures 1B, C**). PD-L1 expression was additionally assessed, revealing a tumor proportion score (TPS) of 68% (**Figure 1D**). Laboratory investigations revealed serum creatinine (Cr) levels ranging from 60 to 99 µmol/L (reference range: 57–111 µmol/L), blood urea nitrogen (BUN) levels ranging from 4.05 to 7.19 mmol/L (reference range: 3.6–9.5 mmol/L), alanine aminotransferase (ALT) levels ranging from 34 to 61 U/L (reference range: 9–50 U/L), and aspartate aminotransferase (AST) levels ranging from 25 to 49 U/L (reference range: 15–40 U/L). These parameters indicate preserved hepatic and renal functional reserve in the patient. Furthermore, to establish a prognostic risk model for advanced renal cell carcinoma (RCC), we analyzed laboratory parameters including lactate dehydrogenase (LDH) levels (range: 188–325 U/L; reference: 114–240 U/L), corrected calcium (Ca) levels (range: 2.01-2.23 mmol/L; reference: 2.1-2.6 mmol/L), hemoglobin (Hb) levels (range: 123–154 g/L; reference: 130–175 g/L), neutrophil (NE) counts (range: 4.32-10.97 × 10^9^/L; reference: 1.8-6.3 × 10^9^/L), and platelet (PLT) counts (range: 160-296 × 10^9^/L; reference: 125-350 × 10^9^/L). Using both the Memorial Sloan Kettering Cancer Center (MSKCC) criteria and the International Metastatic Renal Cell Carcinoma Database Consortium (IMDC) criteria, patients were stratified into the intermediate-risk group by MSKCC criteria and the poor risk group by IMDC criteria at baseline.

**Figure 2:**
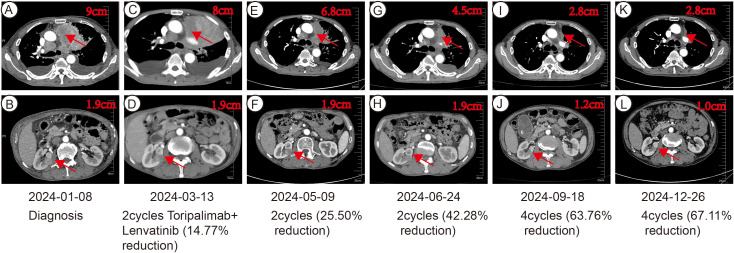
CT imaging assessment of left lung lesion and right kidney mass throughout the course of disease corresponding to their respective time points. **(A, B)** CT image at initial diagnosis on January 8, 2024. **(C, D)** CT image on March 13, 2024. **(E, F)** CT image on May 9, 2024. **(G, H)** CT image on June 24, 2024. **(I, J)** CT image on September 18, 2024. **(K, L)** CT image on December 26, 2024.

**Figure 3:**
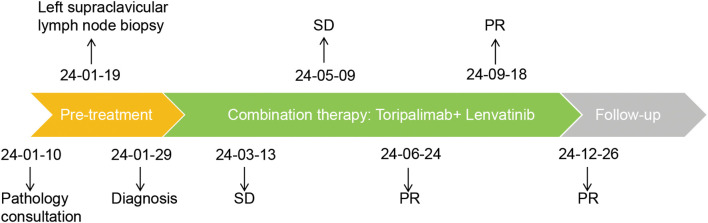
Timeline of the major treatment process and CT evaluation of this case. The tumor response was evaluated according to the criteria defined by RECIST 1.1. PR, Partial Response; SD, Stable Disease.

In January 2024, toripalimab (flat dose of 240 mg every 3 weeks) and lenvatinib (flat dose of 8 mg daily) were initiated. Considering the patient’s financial situation, drug accessibility, and the demonstrated superior efficacy of toripalimab in advanced clear cell renal cell carcinoma (ccRCC) from the phase 3 RENOTORCH clinical trial, toripalimab was selected. At initial diagnosis, the patient was in poor clinical condition, exhibiting symptoms including a productive cough, exertional dyspnea, and bilateral lower limb edema. To optimize treatment tolerance, lenvatinib was initiated at a lower dose of 8 mg. According to the criteria defined by RECIST 1.1, follow-up CT at 3 months demonstrated stable disease (SD), with a 25.50% reduction in the target lesion and resolution of obstructive pneumonia. By September 2024 (10 cycles), metastases in both lungs and right renal nodes regressed further. Efficacy remained PR at 11 months, with no grade ≥3 adverse events (**Figure 2**). Furthermore, the patient’s KPS improved significantly, reaching 90-100. We plan to maintain immunotherapy combined with antiangiogenic therapy until disease progression or the occurrence of intolerable toxicity. A timeline of the major treatment process and CT evaluation of this case is shown in (**Figure 3**).

## 3 Discussion

Type 2 PRCC is characterized by its aggressive nature and poorer prognosis compared to type 1 PRCC (7). The distinct features of type 2 PRCC, including its tendency for lung metastasis and the diagnostic challenges it presents, highlight the urgent need for effective therapeutic strategies. In this case report, we present a 73-year-old male patient with metastatic type 2 PRCC who exhibited a sustained benefit from a combination of toripalimab and lenvatinib, underscoring the potential of this therapeutic approach in managing this complex malignancy.

The diagnosis of metastatic type 2 PRCC can be particularly challenging, especially when it presents as a large lung mass accompanied by respiratory symptoms. In this case, initial diagnostic uncertainty was resolved through a multidisciplinary approach, which included a comprehensive review of pathology slides and re-cutting of specimens. To date, no molecular or IHC markers specific to PRCC have been identified. PRCC typically shows reactivity for PAX8, AE1/AE3, Cam5.2, CD10, vimentin, P504s, and CK7, while demonstrating negativity for CD117 (KIT). Among these markers, P504s and CK7 are the most valuable IHC markers for distinguishing PRCC from other renal tumor types. P504s serves as a sensitive marker for PRCC, although it is not entirely specific (8). CK7 and MET IHC staining was more prevalent in type 1 PRCC and frequently absent (null phenotype) in type 2 PRCC. MET alterations also demonstrate higher frequency in type 1 PRCC. Additionally, type 2 PRCC exhibit Topoisomerase IIα overexpression. Notably, FH deficiency defines a distinct, clinically aggressive subtype that maintains papillary architecture and may histologically mimic type 2 PRCC. This emphasizes the critical importance of accurate histopathological evaluation in diagnosing renal cell carcinoma, particularly in cases where imaging findings are ambiguous. The integration of advanced imaging techniques and expert pathological consultation is essential for ensuring timely and accurate diagnoses, which can significantly impact treatment decisions and patient outcomes.

Currently, there are no established individualized treatment guidelines specifically for type 2 PRCC, with clinical management strategies generally derived from those for RCC. Several phase 2 studies evaluating targeted therapies have been conducted specifically in metastatic PRCC (mPRCC). Those investigated sunitinib (SUPAP) (9), everolimus (RAPTOR) (10), cabozantinib (PAPMET) (11) and axitinib (AXIPAP) (12) as first-line agents. Cabozantinib treatment led to a significantly longer progression-free survival (PFS) period (median 9.0 months, 95% CI 6% to 12%) compared to the sunitinib group (5.6 months, 3% to 7%; HR for progression or death 0.60, 0.37 to 0.97, one-sided p=0.019). The response rate for cabozantinib was 23% versus 4% for sunitinib (two-sided p=0.010) (11). Additionally, axitinib demonstrated promising efficacy in patients with mPRCC, particularly in type 2 PRCC, with manageable toxicity. The progression-free rate at 24 weeks, the primary endpoint, was 45.2% (95% CI 32.6% to not reached), the objective response rate (ORR) was 28.6% (95% CI 15.7% to 44.6%) with 7.7% in type 1 and 35.7% in type 2. The median overall survival (OS) was 18.9 months (95% CI 12.8 to not reached) (12). Furthermore, combination therapies have demonstrated superior efficacy over sunitinib monotherapy across pivotal trials. In CheckMate 9ER (13), nivolumab plus cabozantinib significantly extended median PFS to 16.6 months versus 8.3 months with sunitinib (HR 0.51; P<0.001) and doubled the ORR (55.7% vs. 27.1%; P<0.001). Similarly, in JAVELIN Renal 101 (14), avelumab plus axitinib provided a longer median PFS (13.8 vs. 7.2 months; HR 0.61; P<0.001) and a higher ORR (55.2% vs. 25.5%) in PD-L1-positive patients. Consistent with these findings, the KEYNOTE-426 trial showed that pembrolizumab plus axitinib improved median PFS (15.1 vs. 11.1 months; HR 0.69; P<0.001) and ORR (59.3% vs. 35.7%; P<0.001) compared to sunitinib (15). The clinical efficacy of immunotherapy, either as monotherapy or in combination, has been established in metastatic RCC with a clear cell histologic component (16, 17). However, pivotal studies of ICI excluded non-clear cell renal cell carcinoma (nccRCC). Small retrospective cohorts have reported conflicting results regarding the response of nccRCC to CPI (18–22).

This case illustrates the diagnostic complexity of metastatic type 2 PRCC and the therapeutic potential of combining PD-1 inhibition with antiangiogenic therapy. Initial misdiagnosis as lung cancer underscores the necessity of multidisciplinary collaboration and advanced IHC (e.g., PAX8, P504s) to confirm renal origin (23).

Lenvatinib inhibits tumor angiogenesis via VEGFR/FGFR blockade, while toripalimab enhances antitumor immunity by disrupting PD-1-mediated immunosuppression (24, 25). Synergy between these agents may explain the sustained PR observed here, aligning with recent trials demonstrating improved progression-free survival with TKI-immunotherapy combinations in renal cell carcinoma (13). Interestingly, this case exhibited a high PD-L1 tumor proportion score of 68%, which raises the possibility of its role as a predictive biomarker for response to PD-1 inhibition in nccRCC. Notably, the absence of severe toxicity contrasts with prior reports of combination-related adverse effects, suggesting careful patient selection and monitoring are critical (14). Limitations of the case report include the single-case design and lack of long-term follow-up. We acknowledge that the treatment duration of 11 months is relatively short for assessing long-term outcomes or disease progression. Larger studies are needed to define optimal dosing, duration, and biomarkers for response.

In summary, Toripalimab-lenvatinib combination therapy achieved durable response in metastatic type 2 PRCC with favorable tolerability. This case supports further exploration of dual-targeted strategies in rare, aggressive renal malignancies.

## Data Availability

The original contributions presented in the study are included in the article/**Supplementary Material**. Further inquiries can be directed to the corresponding author.
