# Supplementary figures and images for: Combination therapy with toripalimab and lenvatinib in metastatic type 2 papillary renal cell carcinoma: a Case Report
Source: Front Immunol. 2025 Nov 6;16:1591489. doi: 10.3389/fimmu.2025.1591489 (PMC12631386; doi:10.3389/fimmu.2025.1591489)

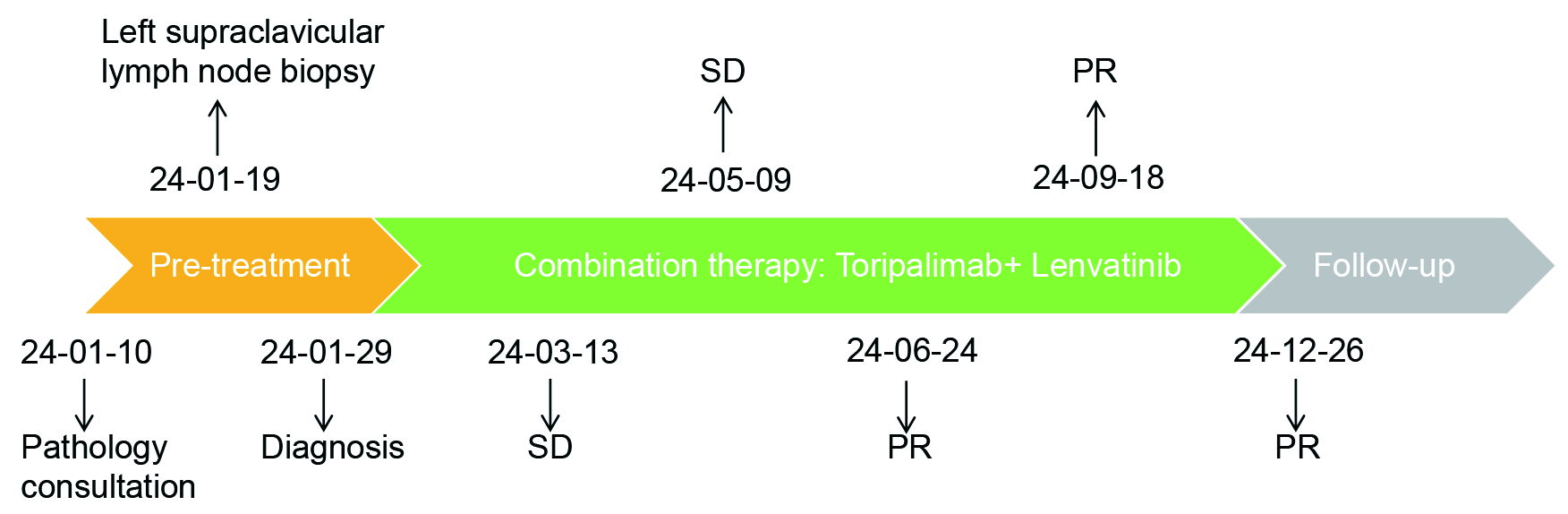

Supplement: Supplementary file 1 [file DataSheet1.zip › Treatment timeline/Figure3.tif]

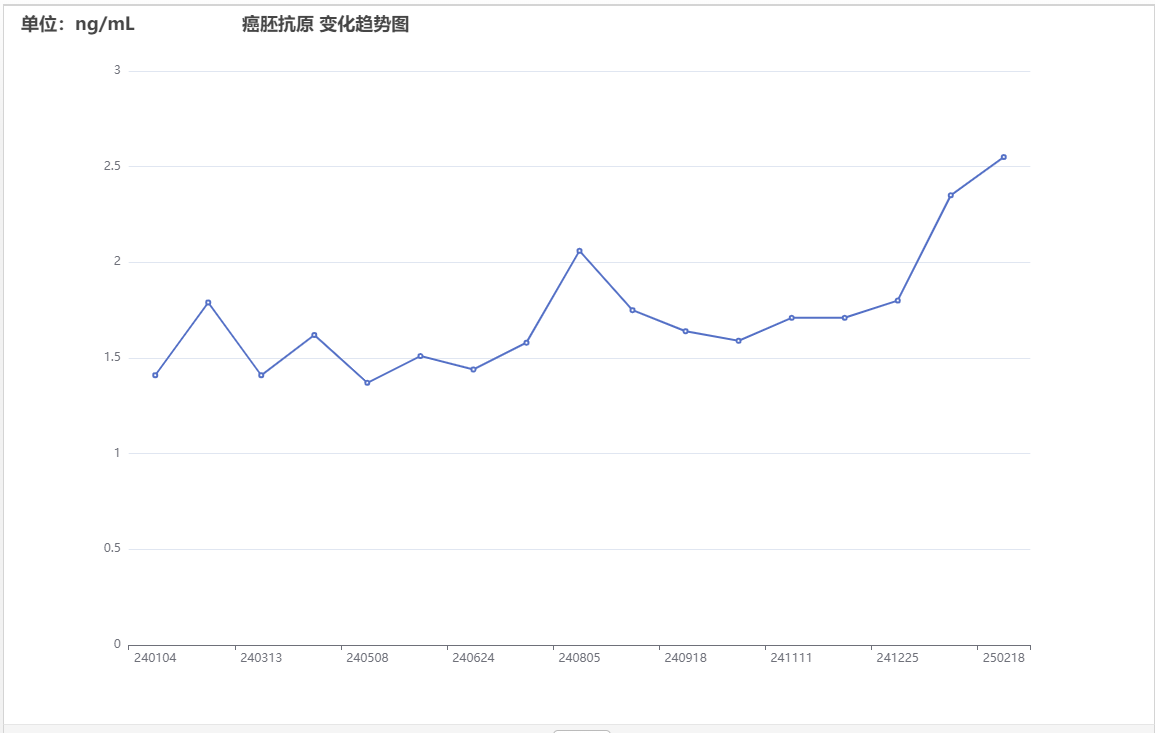

Supplement: Supplementary file 2 [file DataSheet2.zip › Trend chart of tumor markers/Trend of CEA.png]

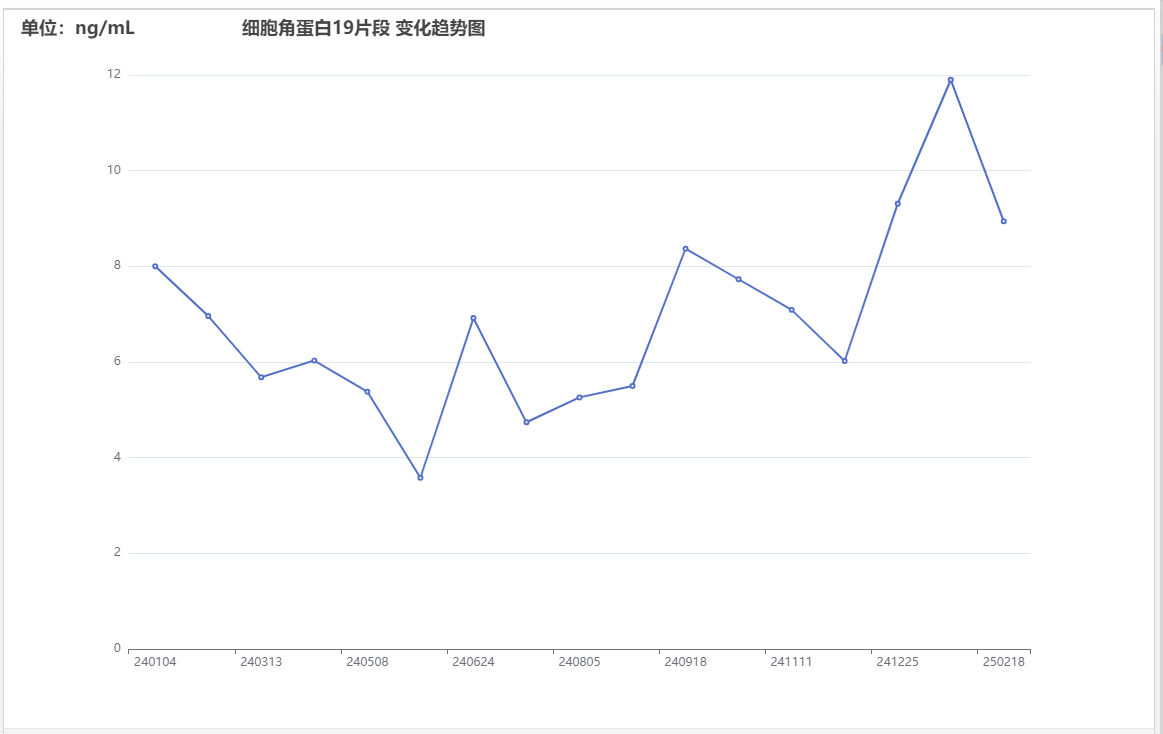

Supplement: Supplementary file 2 [file DataSheet2.zip › Trend chart of tumor markers/Trend of CYFRA21-1.png]

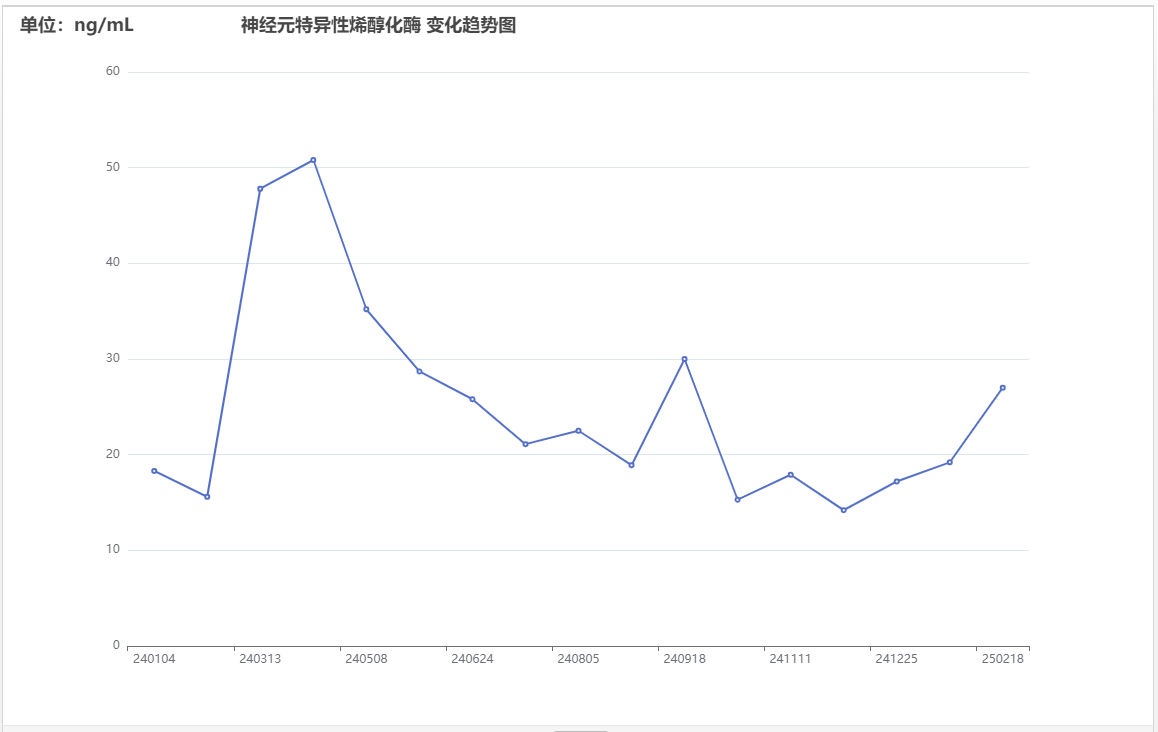

Supplement: Supplementary file 2 [file DataSheet2.zip › Trend chart of tumor markers/Trend of NSE.png]

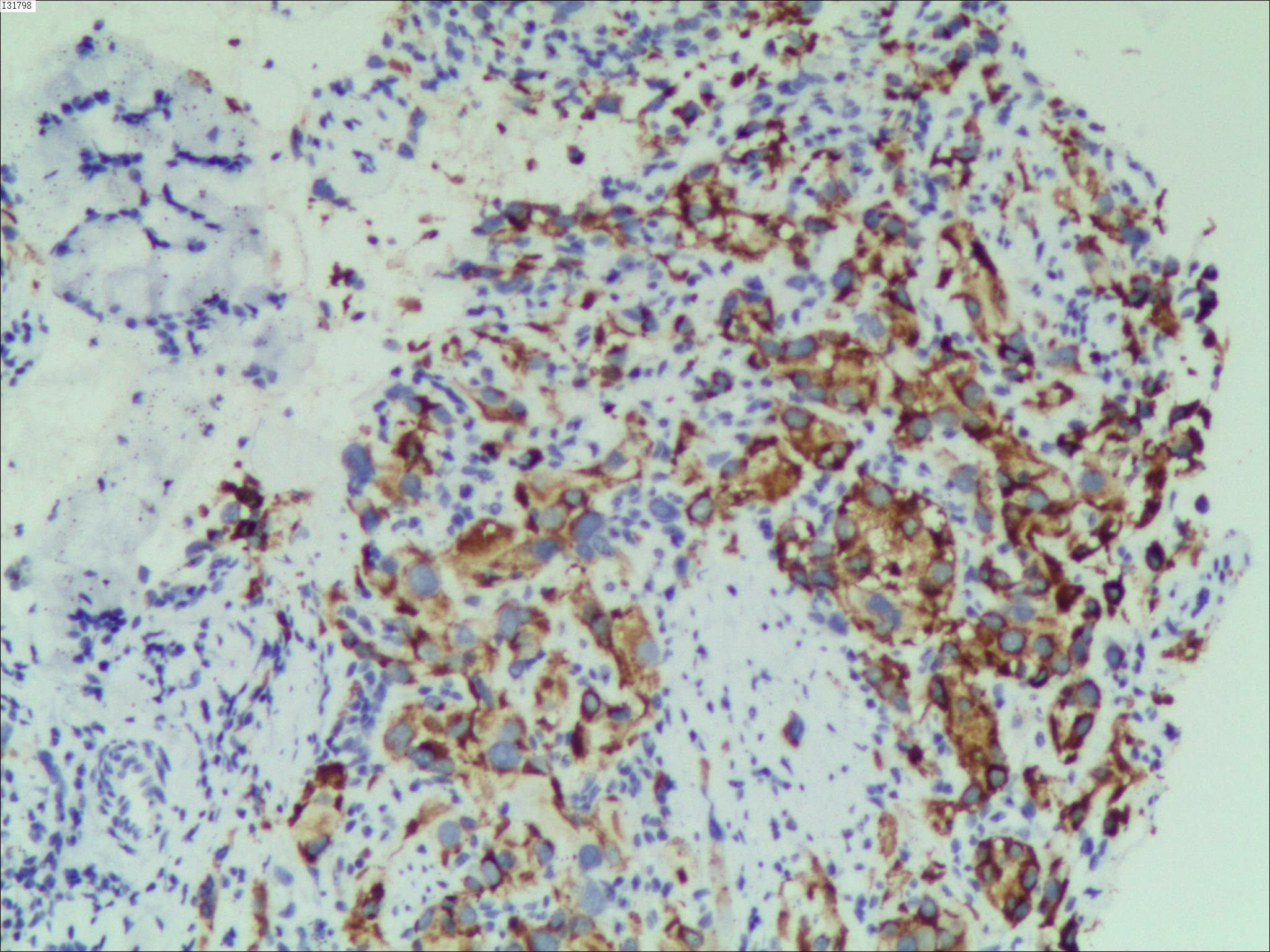

Supplement: Supplementary file 3 [file Image1.jpeg]

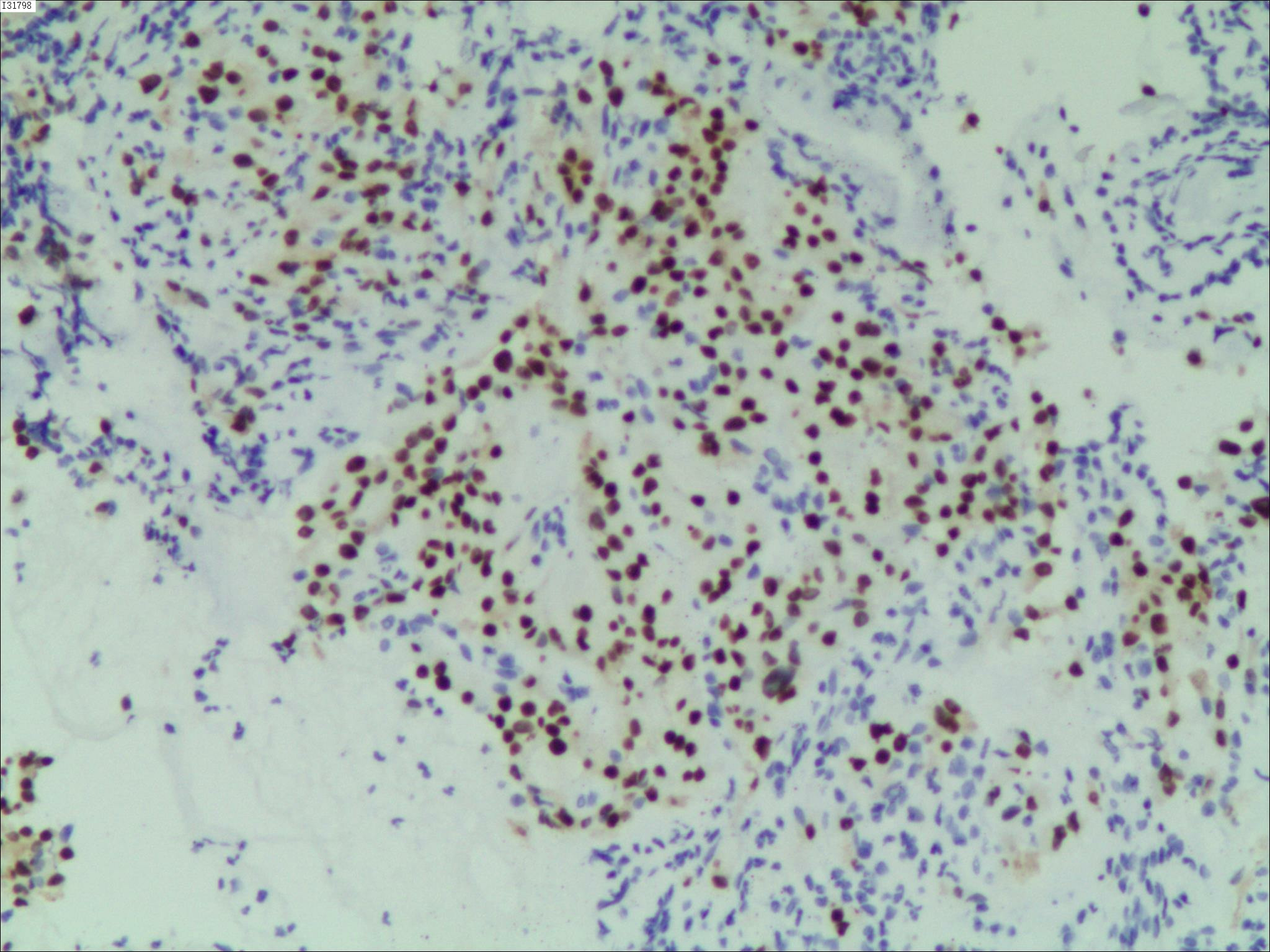

Supplement: Supplementary file 4 [file Image2.jpeg]

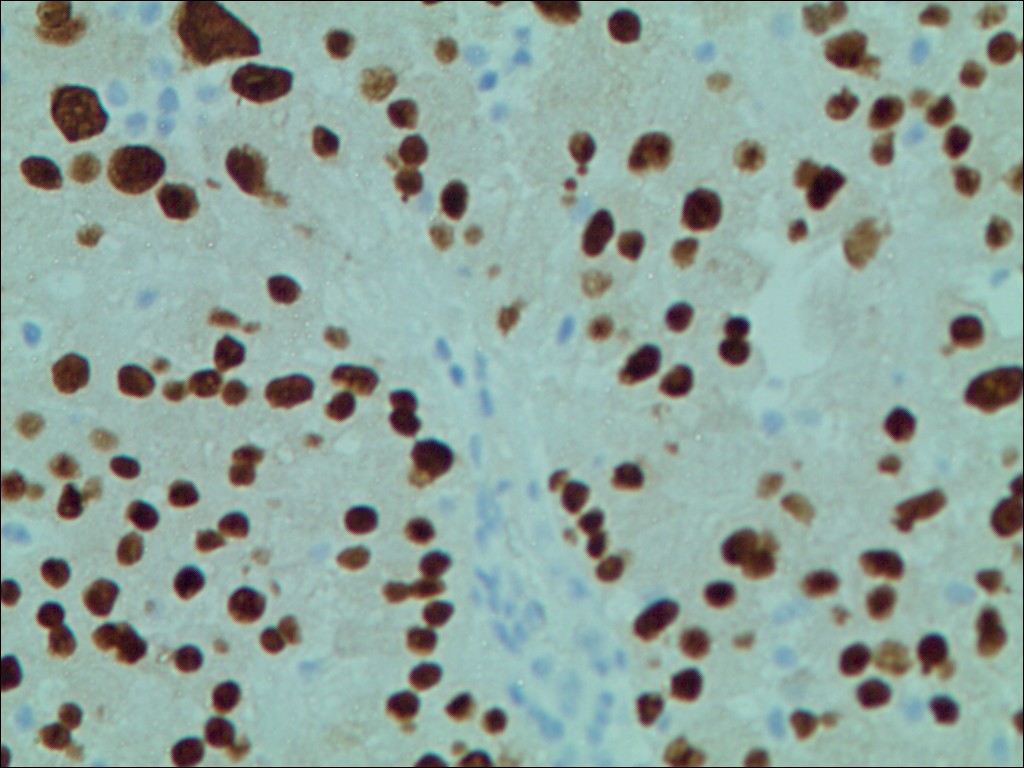

Supplement: Supplementary file 5 [file Image3.jpeg]

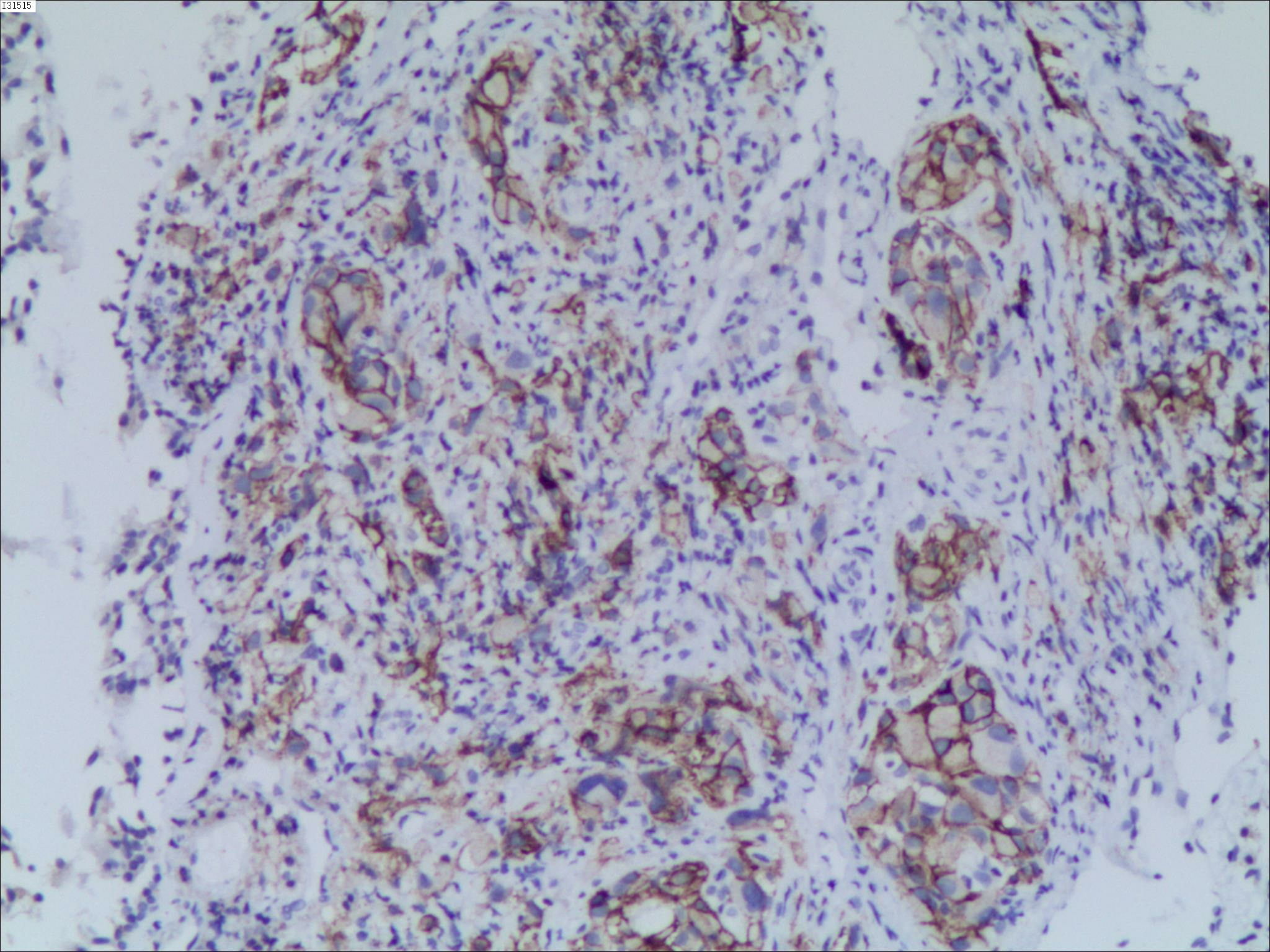

Supplement: Supplementary file 6 [file Image4.jpeg]
